# Knowledge about hypertension and associated factors among patients with hypertension in public health facilities of Gondar city, Northwest Ethiopia: Ordinal logistic regression analysis

**DOI:** 10.1371/journal.pone.0270030

**Published:** 2022-06-17

**Authors:** Maereg Wolde, Telake Azale, Getu Debalkie Demissie, Banchilay Addis

**Affiliations:** 1 Department of Health Education and Behavioral Sciences, College of Medicine and Health Science University of Gondar, Gondar, Ethiopia; 2 Department of Health Policy and Health System, College of Medicine and Health Science University of Gondar, Gondar, Ethiopia; Government College University Faisalabad, Pakistan, PAKISTAN

## Abstract

**Background:**

Hypertension is a disease that imposes risks of diseases on multi-system. Failure to control hypertension leads patients to end up with unavoidable complications, including death. Noncompliance to treatment is the main factor to develop such devastating complications whereas knowledge of patients about their disease is a key factor for better compliance. Thus, the purpose of this study is to assess the level of knowledge about hypertension and associated factors among hypertensive patients in public health facilities of Gondar city.

**Methods:**

Facility-based cross-sectional study was conducted between March and April 2019 in Gondar town. A systematic sampling technique was applied to select a total of 389 patients. A structured interview questionnaire was used to gather the data. The data were analyzed using STATA version 14. Ordinal logistic regression analysis was performed at P < 0.05 with a 95% confidence interval to identify statistically significant variables.

**Results:**

A total of 385 respondents participated giving a response rate of 98.9%. The majority (55.3%) of the patients had a low level of, 17.9% had a moderate level of knowledge whereas 26.8% had a high level of knowledge about hypertension. Those working in government organizations had 5.5 times higher odds of having a high level of knowledge than other groups (AOR = 5.5; 95%CI = 1.21, 25). Patients who received longer than four years of treatment showed twice larger odds of knowledge than those with below two years of treatment (AOR = 2; 95%CI = 1.29, 3.22) Moreover, patients residing proximate to the hospital increases the odds of having a higher level of knowledge by 1.64 times versus patients living far away from the hospital (AOR = 1.64, 95% CI = 1.07–2.63).

**Conclusions:**

This finding revealed that knowledge about hypertension and risk factors among patients with hypertension was low. Employment in governmental organizations, longer duration of treatment, and residential proximity to hospitals/ health centers were statistically significant predictors of the participants’ knowledge about hypertension. Therefore, it is important to give health education to patients working in non-governmental organizations and self-employed individuals about diseases and risk factors. In addition, emphasis should be given to patients receiving less than two years of treatment and coming from remote areas to improve their knowledge of the disease.

## Introduction

Hypertension is a disease that imposes risks of diseases on other systems including on CNS (Central Nervous system), renal system and CVS (Cardiovascular diseases) [1]. Various cardiovascular risk factors have been identified to be a cause of the acquisition of Alzheimer’s disease by affecting the metabolism of cerebral glucose this eventually leads to memory loss and development of diabetes mellitus by causing insulin resistance [2]. Whereas raised Blood Pressure (BP) is among the key risk factors of non-communicable diseases (NCD). NCDs contribute to 71% of all deaths that occur worldwide, and over 85% of these deaths occur in low and middle-income countries [3]. The prevalence is getting higher in lower-income countries including Ethiopia which is estimated to be 19.6% [4].

High blood pressure was associated with being overweight, obese, smoking, some education, the highest wealth index, moderate physical activity, advanced age, and widowhood [5–7]. In addition, lower physical activity, less than five times daily consumption of fruits and vegetables, diabetes, and chronic kidney disease were associated with an increased risk of hypertension [5]. Moreover, a study shows that consumption of certain medicinal plants naturally controls excessive dilatory effects and stop the unwanted reduction of blood pressure like that of antihypertensive treatment [8].

Increased BP is one of the preventable causes of premature deaths. However, most people do not control their blood pressure optimally. Knowledge about increased BP contributes a lot in controlling and preventing the complications it might result [9]. Inadequate understanding of their disease condition in hypertensive patients is making it difficult to control hypertension adequately, in addition, it is one of the leading factors not to adhere to their treatment appropriately [10]. Knowledge about hypertension in hypertensive patients is positively associated with a good adherence level, which in turn helps to control blood pressure [9, 11]. Health education is one approach to provide knowledge which is indicated by a systematic review as it is to help the control of BP, especially in old age group patients [12]. The advantage of their medications and other related issues concerning their medication through health education is expected to result in good blood pressure control and knowledge is believed to be one of the prominent factors to bring good adherence status [13].

Hypertensive patients with good knowledge are scarce in the developing and developed world.

A study done in the USA revealed that about 22% of participants had lower hypertension knowledge [14]. Another study from Brazil showed that 17.7% of participants had little hypertension knowledge and it was significantly associated with non-adherence to antihypertensive treatment [15]. Similarly, a study from Turkey indicates that the majority of participants didn’t have adequate knowledge, in which one-third of the study participants were found to have a low level of knowledge about hypertension [16].

A study conducted in China revealed that about half of the respondents had a low level of knowledge about hypertension [17]. In Pakistan, the level of knowledge among respondents was average in which the respondents hadn’t understood the importance of continuously taking medication well, due to this they were found to be non-adherent [13]. Another study from Pakistan indicated that knowledge about hypertension was limited in patients with hypertension especially those with uncontrolled blood pressure [18]. Similarly, a study from Iran showed that only 25.2% had good knowledge about hypertension [19].

A study done in Zimbabwe found that there is poor hypertension knowledge among hypertensive patients [20]. Similar to this finding a study conducted in Cameroon indicates that there is poor knowledge about hypertension among the patients [11]. Moreover, studies from our country Ethiopia also revealed that there is limited knowledge about hypertension among patients with hypertension [21, 22].

Several studies showed that educational level is significantly associated with knowledge status in which patients with a low level of education were found to have lower hypertension knowledge than their counterparts [14, 16, 19, 20]. An institution-based study done in Pakistan revealed that a low level of hypertension knowledge was common in patients who are unable to control their blood pressure [18].

Studies done from Iran and Ethiopia indicate females and elders have lower hypertension knowledge in comparison to males and younger age groups respectively [19, 22]. Similarly, those with lower income, non-employees, and those who have no regular physical activities were more likely to have lower hypertension knowledge [19]. Moreover, living in rural areas and dietary risk factors were significantly associated with the low level of hypertension knowledge [19, 22].

Patients`compliance with their treatment is affected by numerous factors. Knowledge of patients about their disease is a key factor for better compliance. Good understanding of the level of hypertension knowledge allows designing an intervention that improves the control of raised BP through overcoming misperceptions that guide non-compliance behavior. For better management and control of hypertension, the knowledge status of hypertensive patients should be studied and understood well. However, little is known about the determinant factors of hypertension knowledge of patients on antihypertensive treatment. Therefore, this study aimed to assess the hypertension knowledge level and associated factors among hypertensive patients in public health facilities of Gondar city, Northwest Ethiopia.

## Methods

### Study design and setting

An institution-based cross-sectional study was conducted at four public health facilities (one hospital and three health centers) at Gondar city from March 2019 to April 2019.

### Population

Patients with hypertension who have to follow up at the selected health facilities served as the source population. Adult patients with hypertension who had at least six months of follow-up were included in the study.

### Sample size and sampling methods

The calculated sample size was 389 which were distributed to the four health facilities proportional to the number of patients served at each. The study participants were selected using a systematic random sampling technique.

### Variables of the study

The outcome of this study was knowledge about hypertension and it was classified into three categories low, moderate, and high knowledge. The independent variables of this study were; socio-demographic factors (age, sex, educational status, monthly income, occupation, marital status, and residence), modifying factors (physical exercise, distance from the hospital, social support, and Cost of drugs), and clinical factors (medication schedule, frequency of drug, duration of treatment, co-morbidity, and the number of drugs taken per day).

### Data collection

Data were collected using an interviewer-administered questionnaire that included socio-demographic, psychosocial, and clinical variables. Social support was measured using five items validated chronic illness resources survey (CIRS) [23]. Hypertension-related Knowledge was assessed with 17 items [24]. The questionnaire is a Likert scale with five response categories for each item. The questionnaire was prepared in English and translated to the local language (*Amharic*) for data collection. Data were collected through a face-to-face interview by seven clinical nurses and supervision was made by two health officers. Two days of training were given to the data collectors and supervisors. During data collection, close supervision was made by the supervisor and the investigators checked the filled questionnaires daily and gave on-site corrections.

### Data processing and analysis

The collected data were checked for completeness, coded and entered into EPI-Info version 7software, and exported to STATA version 14 for further analysis. Ordinal logistic regression analyses were carried out to test the association between the outcome and explanatory variables. Model fitness was checked by Hosmer and Lemeshow test which was 0.57.

### Ethics approval and consent to participate

Ethical approval was obtained from the Ethical Review Board of the Institute of Public Health, College of Medicine and Health Science, University of Gondar Ref No/IPH/180/06/2011. Permission letters were obtained from the University of Gondar Referral Hospital. All study participants were oriented on the objectives and purpose of the study before study participation. Confidentiality and anonymity were explained. Patients at health facilities and sick individuals were informed that participation had no impact on the provision of their health care. Study team members safeguarded the confidentiality and anonymity of study participants throughout the entire study. Interviews were conducted in quiet areas, enclosed whenever possible, to ensure participant privacy. To protect the identities of the study participants, each participant was given a unique identification number (ID). Participation in the study was voluntary and individuals were free to withdraw or stop the interview at any time.

## Results

### Sociodemographic characteristics

A total of 385 hypertensive patients participated with a response rate of 98.9%. The mean age was 60(±13) years. Of the total respondents, two hundred twenty-six (58.7%) were females. One hundred eighty-two (47.27%) of them were unable to read and write and one hundred forty-two (56.36%) were farmers. Most of the respondents three hundred thirty-one (85.97%) were urban dwellers (Table 1).

**Table 1:** Sociodemographic characteristics of hypertensive patients in Gondar public health facilities, North West Ethiopia, 2019.

Variable	Description	Frequency(percent)
Age	18–39	25(6.5%)
	40–59	123(31.9%)
	>60	237(61.5%)
Sex	Male	159(43.3%)
	Female	226(58.7%)
Religion	Orthodox	343(89.1%)
	Muslim	40(10.4%)
	Other*	2(0.5%)
Residence	Rural	54(14.1%)
	Urban	331(89.7%)
Occupation	Government employee	55(14.2%)
	Employee in private	51(13.29%)
	Merchant	32(8.31%)
	Farmer	142(36.88%)
	Housewife	95(24.68%)
	Other*	10(2.6%)
Income	<999 ETB	153(39.74%
	1000–1999 ETB	101(26.23%)
	2000–2999 ETB	56(14.55%)
	>3000 ETB	75(19.48%)
Educational status	Unable to read and write	182(47.27%)
	Only read and write	60(15.58%)
	Primary	77(20%)
	Secondary	20(5.19%)
	College and above	46(11.95%)
Marital status	Single	28(7.27%)
	Married	217(56.36%)
	Widowed	94(24.42%)
	Divorced	46(11.95%0

*others in religion = (Protestant, Catholic, and Jewish)

*others in occupation = (Daily laborer, Unemployed, Student, and Retired)

### Clinical and modifying factors

Most (90.13%) of the respondents have been told about proper medication use. Nearly two-thirds (64.68%) cover the expense of their medicine by themselves (Table 2).

**Table 2:** Clinical and modifying factors of the study participants (n = 385) in Gondar public health facilities, Northwest Ethiopia, 2019.

Variable	Description	Frequency/percent
Comorbidity	None	199(51.69%)
	One	107(27.79%)
	More than one	29(20.52%)
Social support	Poor	191(49.6%)
	Good	194(50.4%)
Exercise	No	50(13%)
	Yes	298(77.4%)
	I don’t know	37(9.6%)
Medication schedule	Morning	68(17.66%)
	Evening	153(39.74%)
	Both times	154(40%)
	Three times	10(2.6%)
Frequency of drug	Once	217(56.36%)
	Twice	131(34.03%)
	Three times or more	37(9.61%)
HTN status	Non-controlled	224(41.8%)
	Controlled	161(58.2%)

### Knowledge about hypertension

**Fig 1:**
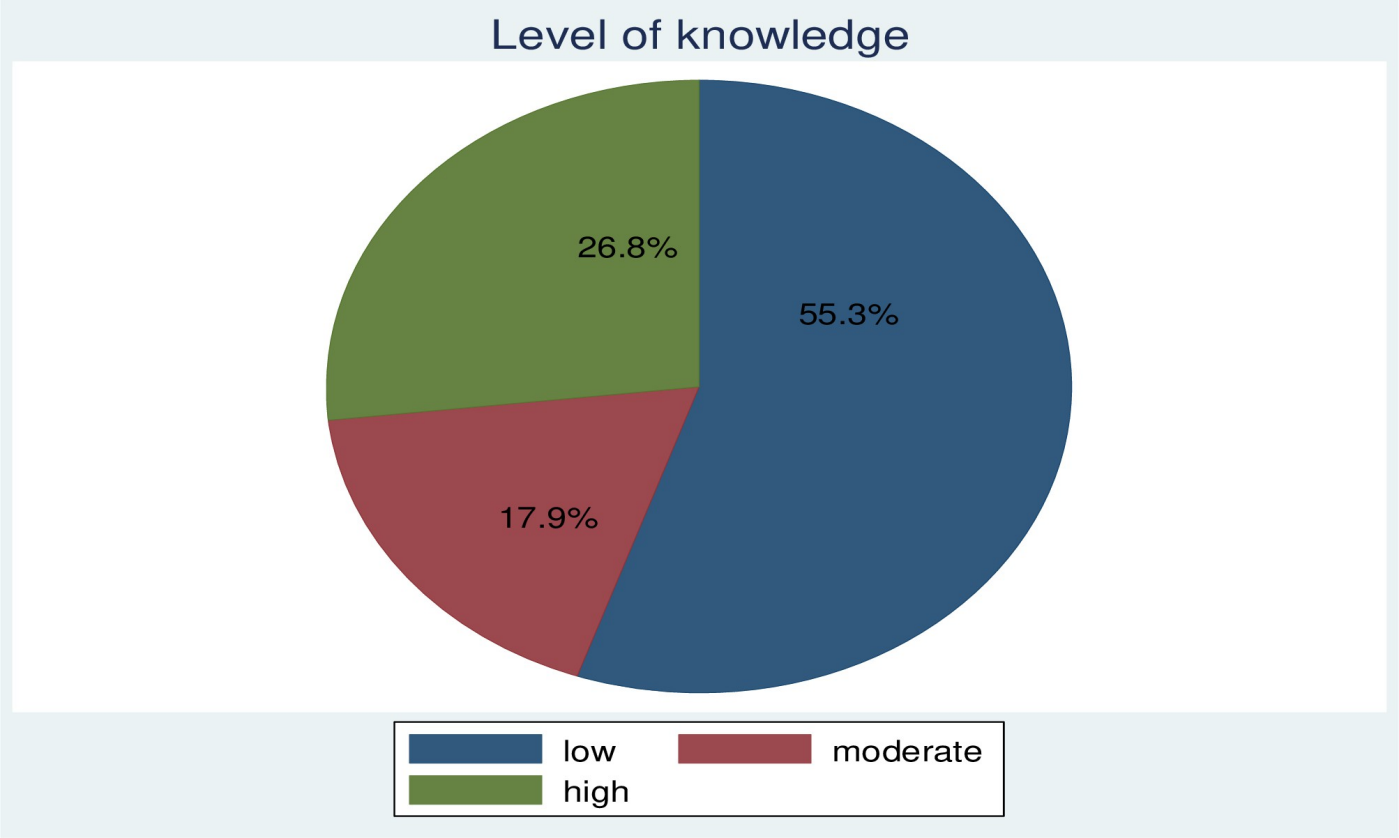
Level of knowledge of the study participants (n = 385) in public health facilities of Gondar town, Northwest, Ethiopia, 2019.

Of the total of 385 participants, 55.3% (213) of them had a low level of, 17.9% (69) had a moderate level of knowledge whereas 26.8% (103) had a high level of knowledge about hypertension (Fig 1).

In the bi-variable regression model sex, cost of drugs, medication schedule, duration of treatment, distance from the hospital, occupation, and comorbidity were significantly associated with a level of knowledge with a P-value <0.2. In the multivariable model occupation, duration of treatment, and distance from the hospital were significantly associated factors.

The odds of having a high level of knowledge among respondents who work at government organizations (versus moderate and low level of knowledge) were 5.5 times higher than all other working groups (AOR = 5.5; 95%CI = 1.21, 25). The odds of having a high level of knowledge among those who were in longer duration of treatment were (relative to moderate and low level of knowledge) 2 times higher as compared with patients with less than two years duration of treatment (AOR = 2; 95%CI = 1.29, 3.22). Moreover, the odds of having a higher level of knowledge among patients who live proximate to the hospital (versus moderate and low level of knowledge) were 1.64 times higher than those who live in a far area (AOR = 1.64, 95% CI = 1.07–2.63) (Table 3).

**Table 3:** Factors affecting Knowledge of HTN among Hypertensive patients in Gondar public health facilities, North West Ethiopia, 2019.

Variable		COR (95% CI)	P-value	AOR (95% CI)
Sex	Male	1		1
	Female	0.69(0.47, 1.03)	0.23	0.7(0.41, 1.24)
Occupation	Government employed	5.6(1.32, 24)	0.03	5.5(1.21, 25) **
	Private employed	1.8 (0.35,8.9)	0.58	1.6 (0.29, 8.62)
	Merchant	2.6 (0.62, 10.6)	0.29	2.2 (0.51, 9.63)
	Farmer	1.5 (0.35, 6.78)	0.72	1.3 (0.28, 6.25)
	Unemployed	1.4 (0.37, 5.56)	0.51	1.6 (0.39, 6.73)
	Retired	2.4(0.62, 9.61)	0.43	1.8(0.42, 7.4)
	Others	1		1
Years of treatment	<2 years	1		1
	2–4 years	1.44 (0.82, 2.54)	0.26	1.41 (0.77, 2.56)
	>4years	1.79(1.17, 2.73)	0.002	2(1.29, 3.22) **
Who covers drug expenses?	Self	1		1
	Free	1.1 (0.69, 1.65)	0.75	0.75 (0.67, 1.74)
	Family	0.9(0.39, 2.23)	0.6	0.6(0.51, 3.2)
	Office	4.5(0.79, 25.5)	0.62	0.62(0.24, 10.6)
Medication schedule	Morning	1		1
	Evening	1.05(0.61, 1.81)	0.67	0.88(0.49, 1.58)
	Both times	0.92(0.53, 1.6)	0.56	0.84(0.47, 1.51)
	Three times	0.14(0.02, 1.16)	0.06	0.13(0.01, 1.11)
How long does it take to come to the hospital?	< = 0.05hour	1.9(1.23–2.95)	0.04	**1.64(1.07–2.63)** **
	>0.05hour	1		1
Presence of comorbidity	None	1		1
	One	0.89(0.57, 1.41)	0.56	0.86(0.53, 1.41)
	Two and above	1.42(0.87, 2.32)	0.39	1.27(0.73, 2.19)

Indicates p value <0.05*

## Discussion

The current study aimed to assess knowledge on hypertension and factors associated factors among patients with hypertension. This study found that about 55.3% of respondents have a low level of knowledge on hypertension and risk factors with 95% CI 50.3% to 60.2%. About 17.9% of them have a moderate level of knowledge with 95% CI 14% to 22% while only 26.5% with 95% CI 23% to 31% of them have a high level of knowledge. Government employees’ long years of treatment and nearer distance from the hospital were the factors significantly associated with a level of knowledge of hypertension.

This study is in line with studies done in Thailand [25]. However, studies done at Uzbekistan, St. Paul Ethiopia, China, Cameroon, South Iran 2, Kwazulu, Srilanka, and North Carolina revealed that there was a better level of knowledge about hypertension than the current study [9, 11, 14, 17, 19, 22, 26, 27]. This difference might be due to socio-economic differences, the difference in knowledge measurement tools, and the inclusion criteria they used to include participants.

However, the level of knowledge of the current study is greater than a study done in Brazil and Pakistan [15, 18]. The possible reason might be the study period difference and socio-demographic discrepancy.

For the second objective, government employees, long years of treatment, and proximate distance from the hospital were significantly associated with the level of knowledge about hypertension as compared to the other groups. Those who work in government were found with a higher level of knowledge (versus moderate and low level of knowledge) than other working groups. This may be because of their higher education level as compared to other groups and this might have a contribution for having a higher level of knowledge while a higher level of educational status was associated with a higher level of knowledge about hypertension and this is supported by studies done in Austria, Ethiopia, Thailand, South Iran, Zimbabwe, and North Carolina [14, 19, 20, 22, 25, 28]. Literate individuals have better information exposure about hypertension through different ways including reading of Information, Education and Communication (IEC) materials prepared to patients with hypertension and this enhances their chance of obtaining adequate knowledge about hypertension.

Patients who were on treatment for longer years duration of treatment were found to have a higher level of knowledge (related to moderate and low level of knowledge) as compared to patients with less than two years of treatment. However, the finding is in contrast with a study conducted in South Africa [26]. The possible explanation for the finding of the current study might be due to patients with a longer duration of treatment having a better chance to hear different information and get advice from health professionals. Also, to attend different health education sessions more about their disease than patients with recent years of diagnosis. Thus, such exposures to various useful information may lead them to be more knowledgeable about their disease.

The result of the present study showed that patients living proximate to the hospital had a higher level of knowledge (compared to moderate and low level of knowledge) than those who live far areas. The possible explanation for this could be the closest distance might allow patients to come to their appointment on time without delay which could increase their chances of obtaining health information from health care workers delivering health education in the early morning in the waiting area.

There are potential limitations of the present study, one of which is a social desirability bias because we used face-to-face interviews, which may lead to social desirability bias and this could overestimate the result. On top of this, there might be recall bias, which might overestimate or underestimate the result. Furthermore, using a cross-sectional study that is able only to detect associations, but no causalities was the other limitation.

## Conclusion

This finding revealed that knowledge about hypertension among patients with hypertension was low. Government employees, a longer duration of treatment, and proximity to the hospital were significantly associated with a higher level of knowledge about hypertension. As a result, health care professionals need to educate patients who are other than a government employee. As well, emphasis should be placed on patients who are on treatment for less than two years and who come from far areas to improve the knowledge of patients with hypertension about the disease.

## Supporting information

S1 File (DTA)

## Abbreviations and acronyms

AOR: Adjusted Odd Ratios
BP: Blood Pressure
CVS: Cardio Vascular System
CNS: Central Nervous System
CIRS: Chronic Illness Resources Survey
ETB: Ethiopian Birr
NCD: Non-Communicable Diseases
USA: United States of America
