# Correction: Knowledge about hypertension and associated factors among patients with hypertension in public health facilities of Gondar city, Northwest Ethiopia: Ordinal logistic regression analysis

**DOI:** 10.1371/journal.pone.0305376

**Published:** 2024-06-06

**Authors:** Maereg Wolde, Telake Azale, Getu Debalkie Demissie, Banchlay Addis

The fourth author’s name is spelled incorrectly. The correct name is: Banchlay Addis. The correct citation is: Wolde M, Azale T, Debalkie Demissie G, Addis B (2022) Knowledge about hypertension and associated factors among patients with hypertension in public health facilities of Gondar city, Northwest Ethiopia: Ordinal logistic regression analysis. PLoS ONE 17(6): e0270030. https://doi.org/10.1371/journal.pone.0270030.

## References

[1] Wolde M, Azale T, Debalkie Demissie G, Addis B (2022) Knowledge about hypertension and associated factors among patients with hypertension in public health facilities of Gondar city, Northwest Ethiopia: Ordinal logistic regression analysis. PLoS ONE 17(6): e0270030. doi: 10.1371/journal.pone.0270030 35714113 PMC9205496

